# Otorhinolaryngological and Ophthalmological Manifestations of COVID-19 in the Pediatric Population

**DOI:** 10.31729/jnma.5450

**Published:** 2020-12-31

**Authors:** Sanju Thapa Magar, Bigyan Raj Gyawali

**Affiliations:** 1 Department of Ophthalmology, AnD Health Services and ENT Care Center, Chandragiri-3, Kathmandu, Nepal; 2 Department of ENT-HNS, Maharajgunj Medical Campus, Institute of Medicine, T.U. Teaching Hospital, Kathmandu, Nepal

**Keywords:** *COVID-19*, *ophthalmological*, *otorhinolaryngological*, *pediatric*, *SARS virus*

## Abstract

Severe Acute Respiratory Syndrome Coronavirus 2 pandemic has affected several countries throughout the world. Being very contagious, it can affect any individual. So far, the prevalence of Severe Acute Respiratory Syndrome Coronavirus 2 in children amongst the total infected population is very low, ranging from 1-5%. Difficulty in diagnosing the disease clinically in the pediatric population owing to their inability to explain their symptoms often renders a possibility of overlooking this disease. Moreover, new modes of presentation are being reported apart from the classical tell-tale signs. In this scenario, medical professionals dealing with the children should be well aware of different modes of presentation of this disease in the pediatric population. This study thus aims to review otorhinolaryngological and ophthalmological manifestations in the pediatric population affected with Severe Acute Respiratory Syndrome Coronavirus 2.

## INTRODUCTION

Severe Acute Respiratory Syndrome Coronavirus 2 (SARS CoV-2) is a highly contagious virus with a worldwide spread affecting a significant number of population. Despite such a rampant spread, the prevalence of SARS CoV-2 in children amongst the total infected population is surprisingly very low, ranging from 1-5%.^1^ This observation has been related to the reduced infection rates owing to the lower expression of Angiotensin-Converting Enzyme 2(ACE-2)receptors in the respiratory epithelium of the pediatric population.^2,3^ Diagnosing the disease clinically in this age group is very challenging as the majority of them cannot explain their symptoms. Also, tell-tale signs of the disease are not always present andnew modes of presentation are being reported. In this scenario, medical professionals dealing with the children have to be well aware of the different modes of presentation of this disease. Here, we review the possible otorhinolaryngological and ophthalmological presentations of SARS CoV-2 in the pediatric population.

## STRUCTURE OF THE VIRUS AND MODES OF TRANSMISSION

A member of the Coronaviridae family, this virus belongs to the b-coronavirus 2b lineage phylogenetically.^4^ This is an enveloped, single-stranded positive-sense RNA virus that has a close resemblance with SARS-CoV and the Middle East Respiratory Syndrome Coronavirus (MERS Co-V). The virus bears spike glycoproteins in its envelope, which after being primed by a protease TMPRSS2, bind with Angiotensin-Converting Enzyme 2 (ACE-2) allowing the entry of the virus into the cell.^5^

Animal to human transmission was initially considered for the emergence of the disease, but now human to human transmission has caused a worldwide pandemic. The basic reproduction number (R0) is thought to be 2.2.^6^ Similar to other respiratory viruses, aerosols tend to transmit the disease, however, the virus has also been detected in tears, conjunctival secretion, urine, blood, and stool.^7,8^

## CLINICAL PRESENTATION

### 1. Age and gender distribution

There has been no specific age group predilection for the disease in children and the minimal age reported is one day.^9^ However, there is a slight male preponderance compared to females with M: F of 1.2-1.8:1(Table 1).^8,10^

**Table 1:** Summary of pediatric otorhinolaryngological manifestations in various studies.

Author(s)	Total cases	Age range	M:F	ENT signs and symptoms (No. of cases)
Song et al.^11^	16	1 month-14 years	1.6:1	Dry cough (3) Cough with fever (3)
Qiu et al.^12^	36	1-3 years	1.7:1	Dry cough (7) Sore throat (2) Pharyngeal congestion (1)
Xia et al.^9^	20	1 day - 14 years 7 months	1.85:1	Dry cough (13) Nasal discharge (3) Sore throat (1)
Zeng et al.^10^	25	1 month - 14 years	1.27:1	Dry cough (11) Nasal congestion (2)

### 2. Ear, nose, throat (ENT) symptoms

#### a. General

General otorhinolaryngological presentation in SARS CoV-2 infected pediatric patients is highly non-specific. In a study by Son et al. in 16 infected pediatric patients, three patients presented with dry cough and three with fever and cough.^11^ In an observational by Quin et al., seven out of 36 patients presented with dry cough and two cases with a sore throat. Pharyngeal congestion was seen in one case.^12^ Similarly, Xia et al. reported dry cough in 13, nasal discharge in three, and sore throat in one of 20 infected pediatric cases. In another study by Zeng et al., of 25 patients, 11 presented with dry cough and two with nasal congestion (Table 1). A nationwide case series by Dong et al. in China including 2135 patients aged 1-18 years showed 51% of cases presenting with mild symptoms.^2^ Although not specified, the majority of cases presented with non-specific URTI symptoms such as fever, cough, sore throat, nasal discharge, and sneezing.

#### b. Symptoms of neural inflammation

Coronavirus has been known to affect neuronal tissue by various mechanisms such as direct injury via neuronal pathways being transported through neuronal proteins such as dynein and kinesin, hypoxic injury, immune injury, and binding to ACE-2.^13^ Netland et al. have a well-documented spread of viral antigen intracranially via olfactory bulb in K18-hACE2 mice model.^14^ While the lungs are the major site of infection, the brain is also infected in some patients. Brain infection may result in long-term neurological sequelae, but little is known about the pathogenesis of SARS-CoV in this organ. We previously showed that the brain was a major target organ for infection in mice that are transgenic for the SARS-CoV receptor (human angiotensin-converting enzyme 2. A cross-sectional survey by Giacomelli et al. showed 33.9% cases with SAS CoV-2 had either a taste or an olfactory disorder and 18.6% had both taste and olfactory disorder.^15^ Similarly, in a study by Mao et al., 5.6% of cases had taste impairment and 5.1% had smell impairment.^16^ These studies predominantly represented the adult population and not much of these symptoms have been reported in the pediatric population. However, with the case reports of Mak et al. in three older children, symptoms such as anosmia and ageusia cannot be ignored and should always raise suspicion of SARS CoV-2 in the pediatric population as well.^17^

Various theories suggesting the possibilities of sensorineural hearing loss have been put forward such as neural inflammation of auditory centers, vasospasm resulting in reduced perfusion to the inner ear, etc.^18^ Although not documented in the pediatric population, there are few cases of sudden sensorineural hearing loss that have been reported in adults with SARS CoV-2, however, its etiological role is yet to be established.^19,20^ Degen et al. have documented cochlear inflammation in MRI of a patient who developed profound hearing loss during the disease.^20^ An observational study by Mustafa et al. also showed a worsening of high-frequency pure tone thresholds and Transient Evoked Otoacoustic Emission (TEOAE) in infected patients when compared with normal healthy adults.^21^

Findings in the above studies were drawn from the adult population. With the paucity of pediatric cases compared to adults, symptoms related to neural involvement in this population have not been mentioned much in the literature. However, considering the evidence mentioned above, there should always be a suspicion if any of the aforementioned clinical symptoms and signs are encountered.

#### c. A rare association with Kawasaki disease (KD)

Children with KD can often present to otorhinolaryngologist with symptoms of stomatitis and cervical lymphadenopathy. Jones et al. have reported an early case of 6 months old female presenting with features of KD.^22^ The child had a fever, limbic sparing conjunctivitis, prominent tongue papilla, a maculopapular rash with swelling of the hands, and lower extremities. She was tested positive for SARS CoV-2. This single case report cannot confer a direct relation with the virus. However, there are studies well documenting the association of respiratory viruses in cases with KD. Chang et al. compared the rate of detection of various viruses between cases with KD and their age and sex-matched controls. Cases with KD had significantly higher viral detection compared to the control group. The detection rate of Coronavirus in KD cases was 7.1% while it was only 0.9% in the controls.^23^ Similarly, Turnier et al. showed positive detection of various respiratory viruses in 41.9% cases with KD. Rhinovirus and Enterovirus were the most commonly encountered ones (28.1%). Various strains of Coronavirus (229E, NL63, OC43) accounted for 3.6% of total cases.^24^

### 3. Ophthalmological findings

There is plenty of evidence suggesting the possibility of ocular involvement in SARS CoV-2.

A study by Ma et al. have reported ocular manifestations in 22.7% of pediatric cases with SARS CoV-2 with 4.2% cases having ocular symptoms as an early presentation.^25^ The reported manifestations were conjunctival congestion, conjunctival discharge, ocular pain, epiphora, and lid swelling. Similarly, in a pediatric case series by Valente et al., 15% of cases had ocular manifestations of viral conjunctivitis.^26^

They also documented the viral shedding via conjunctival secretion through RT-PCR in three cases. Documentation of similar presentation in adults further supports the association of ocular manifestation with SARS CoV-2.^27,28^

The possibility of ocular infection by SARS Co-V 2 is also supported by the fact that ACE 2 receptors have been found in the human retina, cornea, and conjunctiva.^29^ Also, direct inoculation of the virus via aerosol in the conjunctiva and proximity of nasolacrimal duct opening to the upper airway can offer a route for viruses to enter the eye.^30^ In 2004, van der Hoek et al. isolated the fourth human coronavirus HCoV-NL63 from a seven-month-old child suffering from bronchiolitis and conjunctivitis.^31^ This virus was shown to affect the children predominantly.^32^ In the subsequent year, Varbet et al. reviewed the medical reports of 18 patients with HCoV-NL63 in France. Three (17%) cases were found to have presented with conjunctivitis along with respiratory symptoms.^33^ All of those cases were below 15 years. Even in animal models, various subtypes of Coronavirus are known to affect ocular tissue. Fenile Infectious Peritonitis Virus (FIPV), a subtype of Fenile Corona Virus, is capable of inducing granulomatous inflammation and vasculitis in ocular tissues resulting in uveitis, chorioretinitis, retinal detachment, and optic neuritis in cats.^34^

## WAYS FORWARD

Otorhinolaryngological manifestations of SARS CoV-2 such as sore throat, dry cough, and nasal congestion are quite common in the pediatric population. Although rare, Kawasaki disease and symptoms resulting from neural inflammation such as anosmia and sensorineural hearing loss could also be a presentation of the disease. Viral conjunctivitis is the most commonly documented ophthalmological manifestation. As the virus is novel and more facts are yet to be known, every child presenting with the aforementioned otorhinolaryngological and ophthalmological findings should be meticulously evaluated for the possibility of SARS CoV-2.
